# Rationale and design of mDOT-HuA study: a randomized trial to assess the effect of mobile-directly observed therapy on adherence to hydroxyurea in adults with sickle cell anemia in Tanzania

**DOI:** 10.1186/s12874-016-0245-9

**Published:** 2016-10-18

**Authors:** Abel Makubi, Philip Sasi, Mariam Ngaeje, Enrico M. Novelli, Bruno P. Mmbando, Mark T. Gladwin, Julie Makani

**Affiliations:** 1 School of Medicine, Muhimbili University of Health and Allied Sciences, Dar es Salaam, Tanzania; 2 Muhimbili Sickle cell Programme, Dar es Salaam, Tanzania; 3 Muhimbili National Hospital, Dar es Salaam, Tanzania; 4 Department of Medicine, Vascular Medicine Institute, University of Pittsburgh, Pittsburgh, Pennsylvania USA; 5 National Institute of Medical Research, Tanga, Tanzania; 6 Division of Pulmonary, Allergy, and Critical Care Medicine, University of Pittsburgh, Pittsburgh, Pennsylvania USA; 7 Nuffield Department of Clinical Medicine, University of Oxford, London, UK

**Keywords:** Sickle cell disease, Hydroxyurea, Adherence, Medication possession ratio, Randomized trial

## Abstract

**Background:**

Hydroxyurea (HU) has been demonstrated to be efficacious in reducing complications in individuals with sickle cell anemia (SCA) but poor adherence is a barrier. Directly Observed Therapy (DOT) has been shown to improve adherence in various chronic diseases but there is limited data in adults with SCA.

**Methods and design:**

To examine the effect of mobile-directly observed therapy (mDOT) on adherence to HU (mDOT-HuA) in adults with SCA at Muhimbili National Hospital in Tanzania. The mDOT-HuA study is a single centre, prospective, randomized, open label clinical trial. One-hundred individuals with SCA with haemoglobin SS genotype, aged ≥18 years, living in Dar es Salaam, able and willing to record and submit videos electronically will be included. Participants will be divided into two treatment arms; 50 in the standard monitoring (SM) arm will receive mobile phones and fixed dose HU therapy with standard monitoring; 50 in the mDOT arm will receive mobile phones, fixed dose HU therapy with standard monitoring and a mobile directly observed web based medication adherence monitoring system. The primary outcome is the proportion of participants achieving ≥80 % HU adherence compared between the two arms as assessed through medication possession ratio at the end of 3 months of treatment. REDCap, an open source software application will be used to collect data using clinical research forms. The proportions of adherence in the two arms will be compared by Fisher’s exact test. Analysis of outcomes will have performed by both the intention-to treat and per-protocol methods.

**Discussion:**

Should this study become sucessful, it will have the potential for the development of novel strategies for improving HU adherence in SCA.

**Trial registration:**

ClinicalTrials.gov Identifier: NCT02844673, registered on 25^th^t July 2016 (retrospectively registered).

## Background

In Tanzania, sickle cell anemia (SCA) is a major public health priority; reports show that births of individuals with SCA are estimated to be 8000–11,000 annually [1]. It is associated with significant morbidity caused by pain crises, acute chest syndrome, stroke, pulmonary hypertension, leg ulcers and irreversible organ damage. There is a high burden of SCA in Sub-Saharan Africa with high mortality rate especially in children under 5 years of age [2]. Developed countries account for less than 8 % of the disease burden due to interventions such as neonatal screening, pneumococcal vaccination, prophylactic penicillin treatment and hydroxyurea (HU) treatment, thus reducing mortality and morbidity and increasing survival rate [2, 3].

HU, the only disease modifying therapy for SCA, has been demonstrated to be efficacious in reducing complications such as pain crisis and acute chest syndrome and improving survival [4]. HU works primarily by inducing fetal hemoglobin in patients with SCA. Other hematological parameters routinely affected by the use of HU are the mean corpuscular volume, which tends to increase, and the leukocyte and platelet counts, which are expected to decrease [5]. The main clinical outcome of HU use is a reduction of approximately 50 % in the incidence of vaso-occlusive crisis and acute chest syndrome, a reduction in the number of transfusions received by the patient and an improvement in survival [6, 7].

In spite of its significant benefits, HU is, unfortunately vastly underutilized and poorly adhered to because of barriers at the health care system, provider, treatment, socioeconomic, and patient level [8]. Studies conducted in resource-rich settings to assess HU adherence and compliance have shown poor visit and medication adherence measured by pill count, provider reports and pharmacy refills [9–13]. In sub-Saharan Africa HU use has been further limited due to additional barriers related to the relatively high cost of the drug, scarce availability and local challenges related to the monitoring of side effects. To date, there are no studies documenting adherence to HU in sub-Saharan Africa. The main critical gap in knowledge and research about HU adherence and safety is how to assess and tailor interventions according to individual level barriers to adherence and achieve effectiveness of HU in real world situations [14], especially where the environmental factors and adherence assessment techniques that may impact HU compliance are significantly different from those in the developed countries. Details of previous studies reporting HU adherence in SCA are summarized in Table 1.

**Table 1:** Previous studies reporting adherence to HU treatment in SCA

Author, Year	Study design	Sample size and population	Adherence assessment method	% adherence
Thornburg et al., 2010 [12]	Single site, cross sectional	75 (3.5–17.8 years)	Visit adherence, Morisky score, provider report, Pharmacy-refill	82, 84, 77, 49 respectively
Thornburg et al., 2010 [13]	Multisite randomized controlled trial (Baby HUG)	153, 9 months–1.5 years	80 % of liquid medication taken by volume remaining at study visit	89
Ware et al., 2002 [10]	Multisite randomized controlled trial (HUG-KIDS)	53, 5–15 years	Pill count	94
Kinney et al., 1999 [9]	Multisite randomized controlled trial (HUG-KIDS)	84, 5–15 y ears	Pill count	74
Candrilli et al., (2011) [4]	Retrospective insurance claims	312, mean age 21 years	Medication possession ratio	35

Directly observed therapy (DOT) has been shown to improve adherence in multiple clinical trials in diseases such as Tuberculosis (TB) [15] and HIV infection [16]. DOT is more than supervised pill swallowing; it is also a means to provide support and education. Universal DOT for tuberculosis is reported to be associated with a decrease in the acquisition and transmission of resistant tuberculosis [15]. DOT for HIV had shown to have a significant effect on virology, immunologic, and adherence outcomes, although its efficacy was not supported when restricting analysis to randomized controlled trials [16]. DOT for HIV shows greatest treatment effect when targeting individuals with greater risk of non-adherence and when delivering the intervention that maximizes participant convenience and provides enhanced adherence support [16]. Some recent meta analyses [17] have raised questions about the cost-effectiveness of DOT in TB and HIV suggesting that there is a greater need for randomized clinical trials of these approaches.

The use of videophone technology may be a cost-effective alternative to in-home directly observed administration of medications, particularly when long term or life-long therapy are needed, as in the case of HU in SCA [15, 16]. Creary et al., 2014 [3]. reported that electronic DOT is feasible and acceptable and can achieve high HU adherence in a pilot pediatric study in the United States. Further studies are, however, needed to confirm if electronic DOT can improve HU adherence [15–17], especially in the developing countries.

In the study presented herein, we will evaluate the level of adherence, efficacy and safety of fixed dose HU with or without mDOT in adult HbSS patients at Muhimbili National Hospital (MNH) in Tanzania. The study has the potential for the development of novel strategies for improving HU adherence in SCA.

## Methods

### Study design

**Fig. 1:**
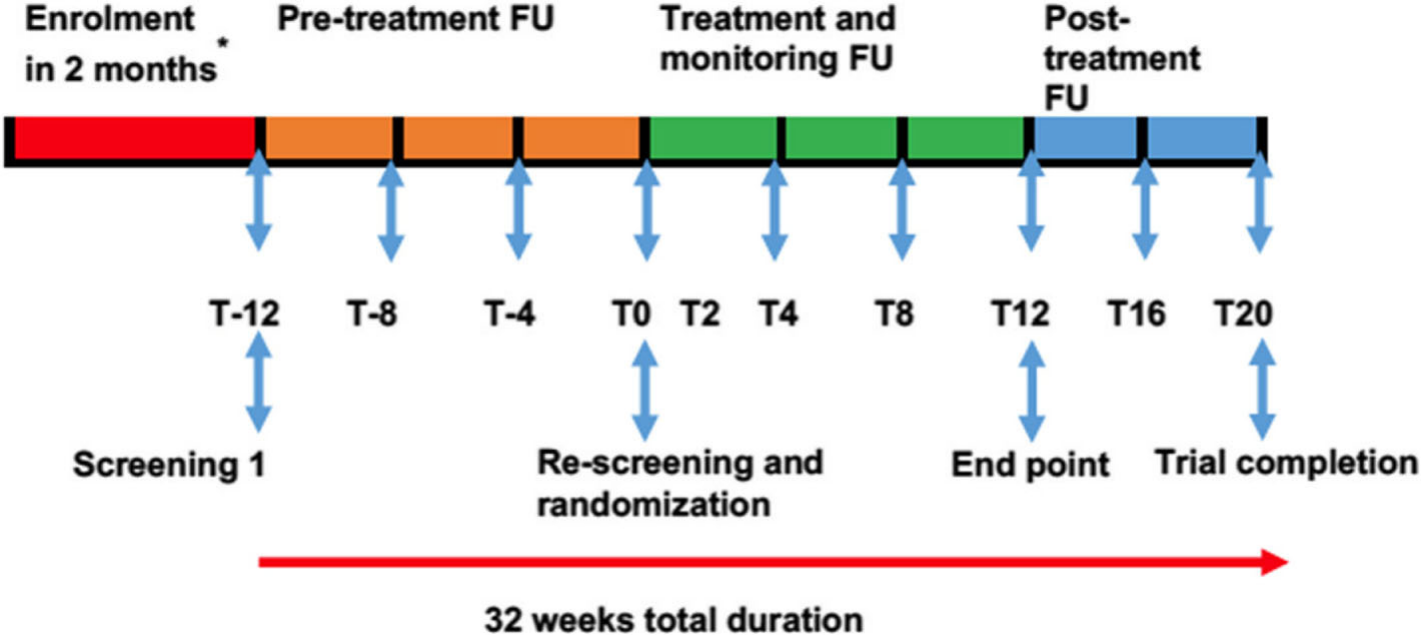
Stages of the trial, events and time points. Keys: *T* time in weeks, − before randomization, *FU* follow-up, * = participants will be enrolled in 2 months but each of them entering at his/her own time and start screening on the same day

The mobile-directly observed therapy (mDOT) on adherence to HU (mDOT-HuA) study is an investigator-initiated, single centre, prospectively designed, randomized, open label phase 4 clinical trial. The study will have four stages: enrolment, pre-treatment follow up, treatment and post-treatment follow up (Fig. 1). Enrolment (2 months) will involve inviting each participant meeting the inclusion criteria below. After each participant has been enrolled, he/she will enter a 3-month pre-treatment follow-up period during which baseline data will be collected. At the end of this period, all patients will be re-screened for eligibility and all those still eligible will be randomized to either arm. After the treatment period, all participants will enter a 2-month post treatment period when they will stop drug intake but continue to be on standard monitoring. Details of the trial design and stages are summarized in the schematic diagram below (Fig. 1).

### Primary and secondary endpoints

The primary endpoint will be the proportion of participants achieving ≥80 % HU adherence as assessed through medication possession ratio (MPR) at the end of 3 months of HU treatment and monitoring. Secondary endpoint will be efficacy of HU treatment as measured through the mean change in fetal hemoglobin (%) at the end of 3 months of HU treatment. Monitoring and safety endpoints will be the proportion of participants experiencing serious adverse events related to HU at week 2, 6, 10 and at the end of 3 months of HU treatment and monitoring.

### Study setting

The study will be conducted at Muhimbili National Hospital (MNH), in Dar es Salaam, Tanzania. MNH serves as the main referral hospital in Tanzania. It houses the Muhimbili Sickle Cell Clinic that serves on average 30 to 60 patients per week.

### Study population

The study population will be drawn from among the approximately 4000 patients with SCA who are registered at the Muhimbili Sickle Cell Clinic. The main mDOT-HuA study inclusion and exclusion criteria are listed in Table 2.

**Table 2:** Summary of the inclusion and exclusion criteria at screening

Inclusion criteria	Exclusion criteria
Hemoglobin SS genotype	Chronic transfusion program as defined by participating in a scheduled (pre-planned) series of transfusions for prophylactic purposes or a hemoglobin A level >20 % of the total hemoglobin
Age ≥18 years and living in Dar es Salaam	Haemoglobin <4.0 g/dL
Male or female (post-menopausal, sterile, or using an acceptable method of contraception)	HIV positive
Negative urine pregnancy test at Screening and a negative urine pregnancy test (dipstick) prior to randomization and dosing	Female planning to become pregnant during the study period
Absolute neutrophil count >1500/uL	Serious mental (including psychosis) or physical illness, which, in the opinion of the investigators would compromise participation in the study (e.g. impaired mental capacity, alcoholism)
Platelet count >95,000/uL	Any condition which the investigators judge to preclude safe participation in the study or to confound the evaluation of the study outcome
Serum creatinine <100 μmol/L (1.2 mg/dL)	
Alanine transaminase less than two times the upper limit of normal	
Being able and willing to record and submit videos electronically.	

### Screening and baseline assessment

This will include the use of database records, interviews and laboratory assessment. At the first clinic visit (T-12, Fig. 1), participant’s eligibility will initially be screened based on data record available from the Muhimbili Sickle Cohort database (i.e. age, sickle cell status) before further work up is done. During further screening/and prior randomization, consenting participants will undergo interviews, laboratory tests including complete blood counts, reticulocyte count, alanine transaminase, lactate dehydrogenase, haemoglobin F, serum creatinine, HIV status, and urine pregnancy test for women and those eligible (answered yes to all inclusion criteria and no to all exclusion criteria) will be included in the trial. The period from enrollment confirmation to the end of 3-month pre-treatment follow-up/re-screening (T-12-T0, Fig. 1) will be used to collect baseline data. At the end of this period, all patients will be re-screened for eligibility and all those still eligible (*n* = 100, planned), (T0, Fig. 1) will enter into the trail.

### Randomization, stratification and blinding

**Fig. 2:**
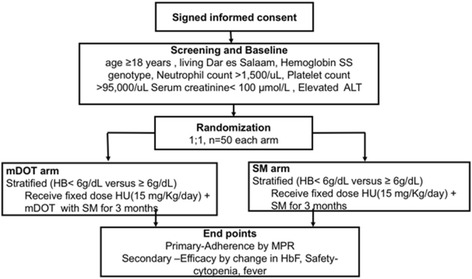
Flow chart for study enrollment and randomization. Keys; *ALT* Alaninie aminotransferase, *SM* Standard monitoring, *MPR* Medication possession ratio

A randomization schedule will be generated using a Research Electronic Data Capture System (REDCap). Participants will be stratified by baseline haemoglobin concentration, just before start of the treatment period (<6 g/dL versus ≥6 g/dL) and assignment of participants to treatment arms will be balanced through the use of a stratified (central) randomization (Fig. 2). This will be an open label trial and participants and investigators will be unblinded.

### Interventions

#### The mDOT arm

Participants will receive mobile phones, fixed dose HU therapy (15 mg/Kg/day) and mDOT consisting of a web based medication adherence monitoring system that includes directly video confirmation of adherence using the patient’s personal cellular telephone (Fig. 2). Participants will receive alerts on their cell phone at pre-arranged times to remind them to take their medications. They will capture a video using their mobile phone of the medication dose and of themselves and the act of swallowing HU. The videos will then be uploaded in the WhatsApp Messenger application (WhatsApp Inc., Mountain View, CA, USA) and sent to the study mobile phone for storage in REDCap. Participants will be followed-up at 2 weeks after initiation of therapy and monthly thereafter.

#### The standard monitoring (SM) arm

Participants will receive mobile phones and fixed dose HU therapy (15 mg/Kg/day) with standard monitoring (Fig. 2). Standard monitoring is defined as a follow-up visit 2 weeks after initiation of therapy and monthly follow-ups thereafter.

### Clinical assessment

During the pre-treatment phase participants will be evaluated for social demographics, clinical symptoms such as painful crises, fever, hospital admission, blood transfusion and measurements of weight, temperature, pulse, and blood pressure will be obtained. After randomization participants will have additional assessment of HU drug adherence and possible adverse drug event.

### Laboratory assessment

This will follow the schedule as indicated in Table 3

**Table 3:** Clinic and laboratory schedule

Clinical/laboratory event	Corresponding visit/time schedule									
	V1	V2	V3	V4	V5	V6	V7	V8	V9	V10
	T-12	T-8	T-4	T0	T2	T4	T8	T12	16	T20
Screening	√									
Visit	√	√	√	√	√	√	√	√	√	√
Vitals	√	√	√	√	√	√	√	√	√	√
Clinical	√	√	√	√	√	√	√	√	√	√
Previous current medication	√									
Past history	√									
Physical Examination	√	√	√	√	√	√	√	√	√	√
Laboratory Sample Collection	√	√	√	√	√	√	√	√	√	√
Inclusion criteria	√			√						

*Abbreviations*: *V* clinic visit, *T* time in weeks, − before randomization

### Laboratory tests

A blood sample (5 ml) will be collected through venipuncture using a vacutainer needle. Two samples will be collected at each bleed: 0.5 ml will be collected in an EDTA tube, well mixed and immediately sent to the Muhimbili Central Pathology laboratory for full blood count and reticulocyte count (determined by automated haematology analyser (Sysmex XT 2000i Kobe, Japan). The second sample, (4.5 ml) will be collected in a heparinized tube, immediately sent to the same laboratory, centrifuged (3000 g for 10 min) and plasma separated into a cryovial for creatinine, Alanine transaminase and Lactate dehydrogenase (Cobas Integra 400 Plus Chemistry Analyzer, Roche, Basel, Switzerland). Haemoglobin F quantification will be determined by high performance liquid chromatography (HPLC) (Bio-Rad Variant I, Bio-Rad, Hercules, CA, USA).

### Assessment of endpoints

*Adherence*: Patients will receive a 30-day supply of HU at each visit during the 3-month treatment period. The study pharmacist will maintain a drug dispensing and prescription record for each patient at the sickle cell clinic. MPR will be calculated at the end of the 90 days of treatment. Adherence will be defined as a MPR ≥0.80.

*Efficacy*: Efficacy will be assessed by measurement of mean change of hemoglobin F between baseline and the end of 3 months.

*Safety*: This is monitored through clinical and laboratory assessment by determining the frequency of fever, malaria and any cytopenia (neutropenia, thrombocytopenia etc. as indicated below in the HU holding and de-escalation criteria; monitoring will be captured at week 2, 6, 10 and at the end of 3 months of HU.

#### Adherence to follow-up clinics and blood tests

A therapeutic alliance between participants and study team and this will maximize scheduled study visit attendance. Since regular blood count is critical in HU therapy, an active follow-up for patients who fail to come for blood count. If it happen some patients did not report to the clinic for laboratory assessments within the predefined intervals, they will not receive subsequent HU refills.

### Subject withdrawal criteria and procedures

Subjects who experience any of the following cytopenias while on HU will be instructed to hold the medication, tested for malaria infection and other intercurrent illnesses and have a laboratory follow-up in 2 weeks. If at the 2 week follow up the cytopenia has resolved and the investigators deem it was due to a transient infectious illness, HU will be restarted at the dose of 15 mg/Kg. If at the 2-week follow-up the cytopenia has resolved and the investigators deem it was due to HU, the subject will receive HU at the de-escalation dose of 10 mg/Kg for the remainder of the study. If at the 2 week follow up there is persistent cytopenia, HU will be stopped and not resumed for the duration of the study, but the subject will continue to undergo standard monitoring, regardless of his treatment arm allocation (Table 4).

**Table 4:** HU holding/de-escalation and stopping criteria

HU holding and de-escalation criteria	HU stopping criteria
Absolute neutrophil count <1500/uL	Positive urine pregnancy test
Platelet count <95,000/uL	Any cytopenia that does not resolve after holding HU for 2 weeks
Hb <9 g/dL with absolute reticulocyte count <95,000/uL	Request of the subject or proxy (withdrawal of consent by the subject)
	Investigator’s discretion
	Subject is lost to follow-up

### Statistical considerations

*Sample size*: A sample size of 50 patients per group provided an 80 % power to detect an estimated proportion with HU adherence of 0.35 in the control group versus 0.65 in the treatment group, assuming a two-sided type I error rate of 5 % and a 15 % dropout rate. The 0.35 proportion of adherence to HU is based on a study done by Candrilli et al. [4].

*Statistical analysis*: The primary outcome of this study is adherence to HU as defined as MPR ≥80 at the end of 3 months of HU treatment. The proportions of adherence in the two arms will be compared by Fisher’s exact test. A forward, stepwise logistic-regression procedure will be then performed to adjust raw adherence difference with use of covariates that will be found to be significant predictors of outcomes. Covariates will be entered into the logistic model at a *P* value of ≤0.10.

***Sub group analysis***: For data analysis, the study population will be categorized into three groups: the intention-to treat (ITT), the Per-Protocol (PP) and the safety populations. These categories will be defined as follows:

*ITT*: All patients who are randomized and receive at least 1 supply of HU, and will be analyzed by randomized treatment group.

*PP*: All ITT patients who complete the study, receive both supplies of HU, and have no major protocol violations that will impact the efficacy assessments. PP population will be documented prior to database lock.

*Safety*: All patients who are randomized and receive at least 1 supply of HU. Safety population will be analyzed by actual treatment received.

## Discussion

There are no published data on the adherence of HU in SCA in African settings and no set gold standard to assess the adherence, effectiveness and efficiency of this therapy in sub-Saharan Africa. Lanzkron et al. conducted a systematic review of HU treatment in adults with SCA and reported that there was a relative increase in HbF from 45 to 20 %, relative reduction in crisis by 68 to 84 % and hospital admissions declined by 18 to 32 %, however lack of long term follow-up limited the conclusion on safety and toxicity [18]. Ware et al. [10] Thornburg et al. [7] and Kinney et al. [9] conducted randomized control trials to address HU adherence in SCA using pill counts, provider report, and pharmacy refills and found that adherence was 94, 89 and 74 %, respectively by the three methods. And all these studies also address a need to conduct of further studies to address adherence to medications using different age groups and approach including DOT. The use of mDOT might have cost implications and patients might only adhere well to drug during observation and go back to the usual behaviors afterwards [19] but with the widespread availability of smartphones and internet use in developing countries like Tanzania [20, 21] this technology may have the potential to overcome some of these barriers. mDOT-HuA study might shed more light on these challenges and provide further information on the post-treatment stage of HU use among individuals with SCA living in Sub Saharan Africa.

## Abbreviations

HIV: Human immunodeficiency virus
HU: Hydroxyurea
mDOT: Mobile directly observed therapy
MDOT-HuA: Mobile directly observed therapy on adherence to Hydroxyurea
MNH: Muhimbili National Hospital
MPR: Medication possession
SCA: Sickle cell anaemia
SM: Standard monitoring
TB: Tuberculosis
